# Use of the rhizobial type III effector gene *nopP* to improve *Agrobacterium rhizogenes-*mediated transformation of *Lotus japonicus*

**DOI:** 10.1186/s13007-021-00764-z

**Published:** 2021-06-23

**Authors:** Yan Wang, Feng Yang, Peng-Fei Zhu, Asaf Khan, Zhi-Ping Xie, Christian Staehelin

**Affiliations:** State Key Laboratory of Biocontrol and Guangdong Key Laboratory of Plant Resources, School of Life Sciences, Sun Yat-Sen University, East Campus, Guangzhou, 510006 China

**Keywords:** *Agrobacterium rhizogenes*, Effector, Hairy roots, *Lotus japonicus*, Plant transformation

## Abstract

**Background:**

Protocols for *Agrobacterium rhizogenes*-mediated hairy root transformation of the model legume *Lotus japonicus* have been established previously. However, little efforts were made in the past to quantify and improve the transformation efficiency. Here, we asked whether effectors (nodulation outer proteins) of the nodule bacterium *Sinorhizobium* sp. NGR234 can promote hairy root transformation of *L. japonicus*. The co-expressed red fluorescent protein DsRed1 was used for visualization of transformed roots and for estimation of the transformation efficiency.

**Results:**

Strong induction of hairy root formation was observed when *A. rhizogenes* strain LBA9402 was used for *L. japonicus* transformation. Expression of the effector gene *nopP* in *L. japonicus* roots resulted in a significantly increased transformation efficiency while *nopL*, *nopM*, and *nopT* did not show such an effect. In *nopP* expressing plants, more than 65% of the formed hairy roots were transgenic as analyzed by red fluorescence emitted by co-transformed *DsRed1*. A nodulation experiment indicated that *nopP* expression did not obviously affect the symbiosis between *L. japonicus* and *Mesorhizobium loti*.

**Conclusion:**

We have established a novel protocol for hairy root transformation of *L. japonicus*. The use of *A. rhizogenes* LBA9402 carrying a binary vector containing *DsRed1* and *nopP* allowed efficient formation and identification of transgenic roots.

**Supplementary Information:**

The online version contains supplementary material available at 10.1186/s13007-021-00764-z.

## Background

*Agrobacterium tumefaciens* (*Rhizobium radiobacter*) causing formation of crown galls and *Agrobacterium rhizogenes* (*Rhizobium rhizogenes*) inducing hairy roots are powerful tools to express genes in plants. In these transformation systems, *Agrobacterium* harbors a plant expression vector (binary vector) with a given gene construct [1]. Like with *A. tumefaciens,* transfer DNA (T-DNA) flanked by defined border sequences is translocated by *A. rhizogenes* into host cells and then integrated into the plant genome [2]. *A. rhizogenes* has been widely used to obtain composite plants that express genes in formed hairy roots. These roots can either be transgenic (i.e., express genes derived from the binary vector) or non-transgenic. Thus, detection of transgenic hairy roots is facilitated by co-transformation of a marker gene that allows visualization of transgenic roots.

Various protocols for *A. rhizogenes*-mediated hairy root transformation of *Lotus japonicus* have been published previously [3–12]. However, relatively little efforts were made in the past to quantify and optimize hairy root transformation of this plant. *L. japonicus* is a model legume widely used to investigate plant development and interactions with nitrogen-fixing bacteria, mycorrhizal fungi and nematodes. The early-flowering ecotype MG20 is particularly suitable for indoor handling [13]. The genome of *L. japonicus* has been completely sequenced [14, 15]. *L. japonicus* plants have been successfully used in various gene mapping studies. Most notably, genes of *L. japonicus* mutants were identified that play a crucial role in the nodule symbiosis between legumes and nitrogen-fixing rhizobia. Nod factor receptor genes, for example, were firstly identified and characterized in *L. japonicus* [16, 17]. Rhizobial Nod factors are lipo-chitooligosaccharidic signals to trigger nodulation signaling in host legumes [18, 19].

In addition to Nod factors, host specific nodule formation often depends on rhizobial effector proteins. Many rhizobia possess a bacterial type 3 secretion system (T3SS) to deliver effector proteins (T3 effectors) into host cells via a needle-like pilus [20]. Proteins secreted by rhizobial T3SSs are generally referred to as nodulation outer proteins (Nops). T3 effectors can influence establishment and maintenance of the nodule symbiosis, presumably by interfering with the plant immune system [20–24]. *Mesorhizobium loti* strain MAFF303099, for example, produces T3 effectors that influence nodule formation of various *Lotus* species [25–28].

In the broad-host-range strain *Sinorhizobium* (*Ensifer*) sp. NGR234, four T3 effectors have been functionally and biochemically characterized [20]. NopL is a protein kinase substrate and interferes with mitogen-activated protein kinase signaling to suppress expression of plant defense genes [29–32]. NopM is a rhizobial E3 ubiquitin ligase that manipulates the plant’s ubiquitin system [33, 34]. NopP is a substrate for unknown plant protein kinases [35]. NopT functions as an effector protease and possesses autocleavage activity [36, 37].

Pathogenic bacteria also often possess a T3SS to translocate T3 effectors into plant cells. AvrPto of *Pseudomonas syringae*, for example, is an effector that targets plant immune receptors, thereby suppressing plant defense responses [38, 39]. *A. tumefaciens*-mediated transient transformation was increased in *AvrPto* expressing *Arabidopsis thaliana* plants*,* suggesting that suppression of plant defense responses stimulated the susceptibility to *Agrobacterium* [40]. We therefore wondered whether rhizobial T3 effectors can be used to promote hairy root transformation of *L. japonicus*. We examined four effectors of *Sinorhizobium* NGR234 and found that *nopP* expression stimulated formation of transgenic hairy roots. Finally, we show that *nopP* expressing roots can be well nodulated by *Mesorhizobium loti*.

## Methods

### Bacterial strains and binary vectors

Used plasmids and bacterial strains are listed in Additional file 1: Table S1. *Escherichia coli* DH5α was grown in LB medium (5 g L^−1^ yeast extract, 10 g L^−1^ tryptone, 10 g L^−1^ NaCl; pH 7.0), *Agrobacterium rhizogenes* LBA1334 [41], K599 [42], and the recently sequenced strain LBA9402 [43] were grown in YMB medium (0.2 g L^−1^ MgSO_4_·7H_2_O, 0.5 g L^−1^ K_2_HPO_4_, 0.1 g L^−1^ NaC1, 2 g L^−1^ mannitol, 0.4 g L^−1^ yeast extract, 15 g L^−1^ agar; pH 7.0). *Sinorhizobium* and *Mesorhizobium* strains were cultured in TY medium (3 g L^−1^ yeast extract, 5 g L^−1^ tryptone, 0.5 g L^−1^ CaCl_2_·2H_2_O; pH 7.0). For hairy root transformation, pISV2678 constructed by Dr. Michael Schultze (University of York, UK) was modified. This binary vector is a derivative of pGPTV-BAR [44] containing a double cauliflower mosaic virus (CaMV) 35S promoter and a translational enhancer sequence from pBI-426 [45]. To visualize transformed roots, *DsRed1,* encoding a red fluorescent protein of the mushroom coral *Discosoma* sp., was used as a transformation marker. The coding sequence of *DsRed1* was PCR-amplified from pX-DR [46] using primers listed in Additional file 1: Table S2. The amplicon was inserted with XhoI and XbaI into pRT104 containing a CaMV 35S promoter and a poly(A) terminator [47]. The expression cassette was then excised with HindIII and cloned into the single HindIII site of pISV2678. The resulting binary vector, named pISV-*DsRed1*, was completely sequenced (GenBank accession number MW701373). The plasmid was then further modified to obtain binary vectors with effector genes driven by the enhanced double CaMV 35S promoter. The T3 effector genes *nopP, nopM, nopL,* and *nopT* were PCR-amplified using genomic DNA of *Sinorhizobium* sp. NGR234 (GenBank accession number U00090.2) as a template. Bacterial DNA isolation was performed as described [48]. Primers of the PCR reactions are shown in Additional file 1: Table S2. The PCR amplicons were then cloned into the multiple cloning site of pISV-*DsRed1* using primer-specific restriction enzyme sites (ClaI, EcoRI and SacI). The resulting vectors were named pISV-*DsRed1*-*nopL*, pISV-*DsRed1*-*nopM*, pISV-*DsRed1*-*nopP*, and pISV-*DsRed1*-*nopT*.

For mobilization of binary vectors into *A. rhizogenes*, 0.5 μg vector DNA was added to 100 μL of pre-cooled competent cells in a sterile 0.2-cm electroporation cuvette. The electroporator (Ding Guo, Guangzhou, China) was set to 1500 V and 10 ms. After eletroporation, 1 mL of YMB medium (kept at room temperature) was added to the cuvette. The bacteria were transferred to 1.5-mL test tubes, manually mixed and then placed on a shaker (200 rpm, 27 °C). After incubation for 3 h, 100 μL of the bacterial suspensions were spread on YMB agar plates containing 100 mg L^−1^ kanamycin. The plates were incubated at 27 °C for two or three days. Finally, single colonies were selected and the presence of plasmids was confirmed by PCR tests using primers shown in Additional file 1: Table S2.

### Plant material and seed germination

Seeds of *Lotus japonicus* (Regel) Larsen ecotype MG20 (Miyakojima MG20; [13]) were soaked in concentrated sulfuric acid for 10 min and washed with water for at least five times. The seeds were then incubated in 70% ethanol for 2 min and transferred to a tenfold diluted commercial bleach solution (~ 0.35% active chlorine; Langqi, Guangzhou, China). The seeds were vigorously shaken on a vortex mixer every 2 min for a period of 10 min. After five-times washing with distilled water, the surface sterilized seeds were evenly suspended on distilled water and incubated overnight at 4 °C. The seeds were then placed on 1.0% (w/v) water agar plates (~ 50 seeds per Petri dish; 15 cm in diameter), which were incubated at an angle of ~ 80° in the dark chamber of a temperature-controlled growth room (24 ± 2 °C). After 3 days, the plates with germinated seedlings were exposed to light/dark conditions (16-h photoperiod; 2000 lx light intensity; Philips Lifemax TL-D 36 W/54-765 and TL-D 36 W/29–530 daylight fluorescent tubes at a ratio 3:1). After incubation for 4 days, seedlings with unfolded cotyledons (green in color) were used for hairy root transformation (~ 80% of germinated seedlings; germination rate ≥  50%).

### Hairy root transformation

Transformation of *L. japonicus* ecotype MG20 was performed with a given *A. rhizogenes* strain carrying pISV-*DsRed1* or pISV-*DsRed1* containing an effector gene. All described steps were performed under sterile conditions. The bacteria were grown on 1.5% (w/v) YMB agar plates supplemented with kanamycin (100 mg L^−1^) and rifampin (25 mg L^−1^). After incubation for 60 h at 27 °C, the bacteria were used for inoculation of germinated seedlings with unfolded cotyledons. Roots of the seedlings were diagonally cut off by a scalpel. The wounded seedlings were then completely dipped into the *A. rhizogenes* colonies. After incubation at room temperature for 30 min in the dark, the inoculated seedlings were placed on agar plates prepared as follows: Round Petri dishes (15 cm in diameter) were filled with 0.9% (w/v) technical agar (HKM, Guangzhou, China) containing 1/2 strength Gamborg’s B5 Salts and Vitamins medium (Sigma-Aldrich) supplemented with 200 μM acetosyringone (HKM, Guangzhou, China). A half of the solid agar was removed to provide more space for developing shoots. The agar plate with the seedlings was then covered with a filter paper of the same size (Whatman, Hangzhou, China) to stabilize the seedlings and reduce formation of condensate water. Each Petri dish contained 10 seedlings placed in a row (in the middle of the plate). The plates were sealed with parafilm with several incisions (using a sterile scalpel to allow air exchange) and incubated in the dark (23 ± 2 °C) at an angle of ~ 80°. All plates were partially covered with aluminum foil to protect the roots from light.

The following day, the plates with seedlings were transferred to the growth room and incubated at light/dark conditions as mentioned above (24 ± 2 °C; angle of ~ 80°). The plates were placed into dark 30 × 12 × 5 cm plastic boxes (without lid) to protect the roots from light effects. Every 7 days, seedlings were transferred to freshly-made agar plates as described above. Plants with formed hairy roots were analyzed 28 or 45 days post inoculation (dpi) with agrobacteria.

### Effector gene expression analysis

Quantitative real time PCR (qRT-PCR) was performed to analyze expressed effector genes in formed hairy roots (28 dpi). Total mRNA (in triplicates) was extracted by an RNA extraction kit following the manufacturer’s instructions (Takara, Tokyo, Japan) and treated with RNase-free DNase (Takara, Tokyo, Japan). The first-strand cDNA was synthesized using the HiScript II Q RT SuperMix for qPCR Kit (Vazyme, Nanjing, China). Reactions (in triplicate) were performed using the LightCycler® 480 SYBR Green I Master Mix in a LightCycler 480 System (Roche Diagnostics, Mannheim, Germany). Primers are listed in Additional file 1: Table S2. Primers of the constitutively expressed *Ubiquitin* gene of *L. japonicus* (GenBank: DQ249171) were used as a reference to normalize the transcript abundance values. Each PCR reaction consisted of 5 μL of cDNA template (500 ng), 10 μM of each primer and 2 μL of the SYBR Green I Master Mix in a final volume of 10 μL. Following thermocycling conditions were used: (i) denaturing: 95 °C for 2 min; (ii) 30 cycles: 95 °C for 30 s, 60 °C for 20 s, 72 °C for 20 s; (iii) melting curves: 95 °C for 30 s, 60 °C for 20 min; (iv) 72 °C for 5 min. Threshold cycles (C_T_ values) were calculated with the Roche LightCycler 480 software. According to the manufacturer’s suggestion, a threshold of 0.1 was defined as the C_T_ detection limit value. Relative gene expression levels were calculated using the 2^−ΔΔCt^ method [49].

### Microscopic analysis

Roots transformed with pISV-*DsRed1* and derivatives were microscopically analyzed for red fluorescence using a Zeiss fluorescence microscope ImagerZ1 (Carl Zeiss AG, Oberkochen, Germany) or a Lumar V12 fluorescence stereo-microscope (Carl Zeiss AG). Red fluorescence conditions were used to distinguish between red fluorescent and non-fluorescent roots. All plant material was photographed under bright field and red fluorescence conditions as recommended by the user manuals of the microscopes. The computer software AxioVision Rel. 4.8 was used to record the pictures. Where indicated, the degree of root tissue showing red fluorescence was determined by the gridline intersection method originally established to quantify the degree of mycorrhizal root colonization [50]. Material from the whole hairy root system was randomly picked and dispersed in plates containing grid lines. Intersections between the grid lines and roots were designated as red fluorescent or non-fluorescent. Root samples from each plant were counted three times. The proportion (%) of red fluorescent tissue was calculated for each plant.

### Nodulation test

Nodule formation was compared for hairy roots transformed with pISV-*DsRed1* and pISV-*DsRed1*-*nopP*. A rifampicin-resistant mutant of *M. loti* strain MAFF303099 was used as an inoculum. Bacteria were grown in liquid TY medium containing 25 mg L^−1^ rifampicin (27 °C, 200 rpm). Bacterial suspensions were centrifuged at 4000 *g* for 10 min and then re-suspended in 10 mM MgSO_4_ (OD_600_ ≈ 0.2). Four weeks after *A. rhizogenes* transformation, transgenic plants (showing red fluorescence in at least one hairy root) were transferred into sterilized 300-mL plastic jar units linked by a cotton wick (1 plant per jar unit). The upper jar contained a mixture of vermiculite and expanded clay (3:1, v/v) and the lower jar was filled with ¼ strength B&D nutrient solution (250 μM CaCl_2_·2H_2_O, 125 μM KH_2_PO_4_, 2.5 μM Fe-citrate, 62.5 μM MgSO_4_·7H_2_O, 375 μM K_2_SO_4_, 0.25 μM MnSO_4_·7H_2_O, 0.5 μM H_3_BO_3_, 0.05 μM CuSO_4_·5H_2_O, 0.025 μM CoSO_4_·7H_2_O, 0.025 μM Na_2_MoO_4_·2H_2_O, 0.125 μM ZnSO_4_·H_2_O) supplemented within 1.0 mM KNO_3_. Each plant was inoculated with 2 mL of the prepared MAFF303099 suspension. The plants were kept under growth room conditions as described above and harvested 4 weeks later.

### Statistical analysis

Data were acquired for each plant and expressed as mean ± SE (n = number of plants). For RNA extraction, 5 plants were combined (n = number of RNA samples). Data were statistically analyzed using Excel and SPSS software. After Analysis of Variance (ANOVA), pairwise comparisons were performed using Duncan’s Multiple Range test with a significance threshold of 0.05.

## Results

### Hairy root formation induced by *A. rhizogenes* LBA9402 carrying pISV-*DsRed1*

**Fig. 1:**
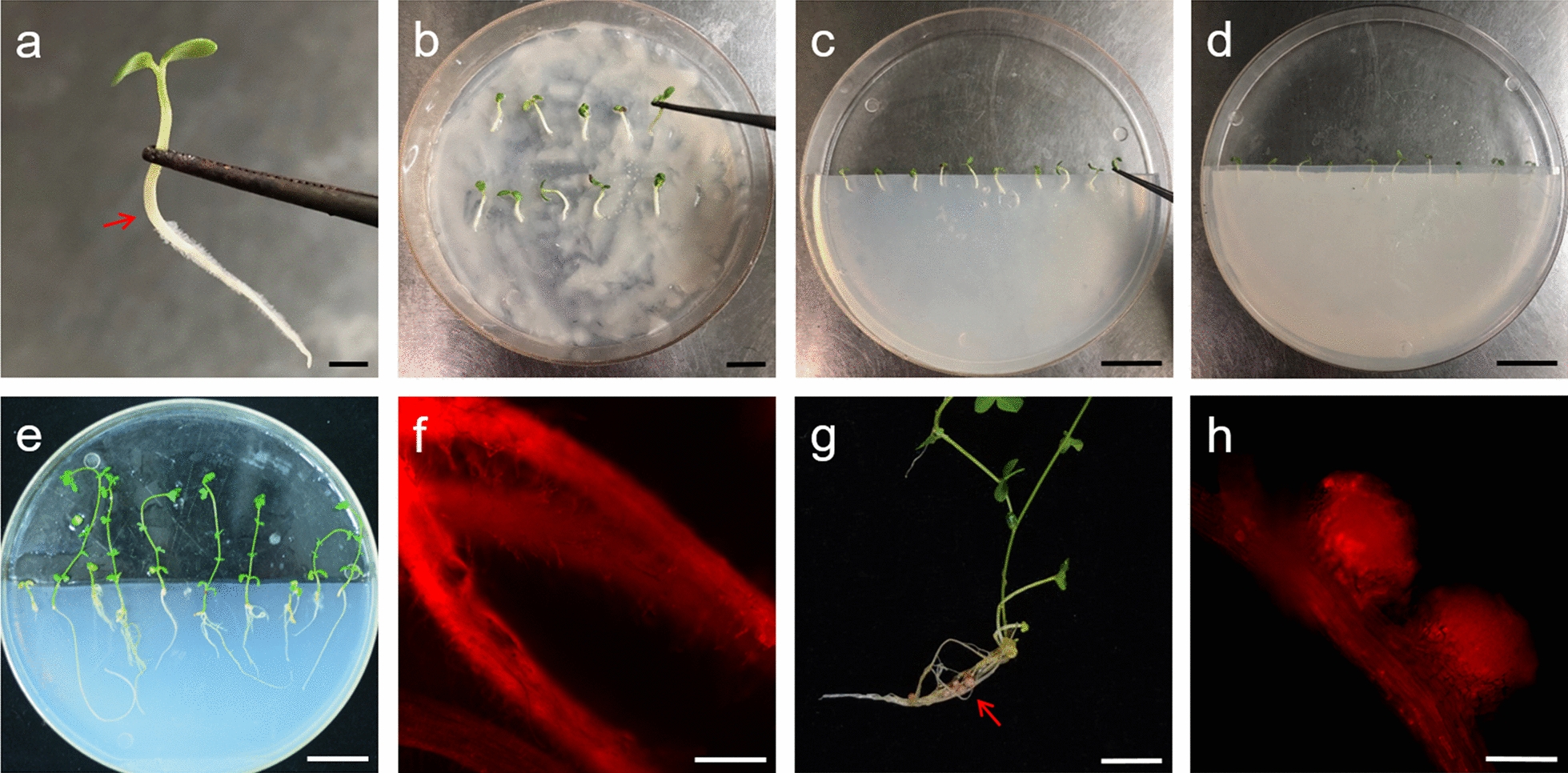
Pictures illustrating the described transformation procedure. **a** Roots from 5-day-old *L. japonicus* MG20 seedlings were removed. The arrow indicates the cutting site at the bottom of the hypocotyl. Seedlings were co-cultivated with *A. rhizogenes* for 30 min. **c** Seedlings were transferred to agar plates containing ½ strength Gamborg’s B5 Salts and Vitamins medium. **d** The agar with seedlings was covered with a sterile filter paper and the plate sealed with parafilm. **e** Plants were weekly transferred to a fresh agar plate and covered by a new filter paper. **f** Analysis by fluorescence microscopy (28 dpi): Selected plants showing at least one red fluorescent root were placed into plastic jar units and inoculated with *M. loti* MAFF303099. **g** Plants with formed nodules (arrow) were harvested 4 weeks later. **h** Analysis of roots and nodules by fluorescence microscopy. The picture shows a red fluorescent root with two nodules. Bars = 1 mm in **a**, 1 cm in **b**, 2 cm in **c**–**e**, 500 μm in **f**, 2 cm in **g**, and 500 μm in **h**

**Fig. 2:**
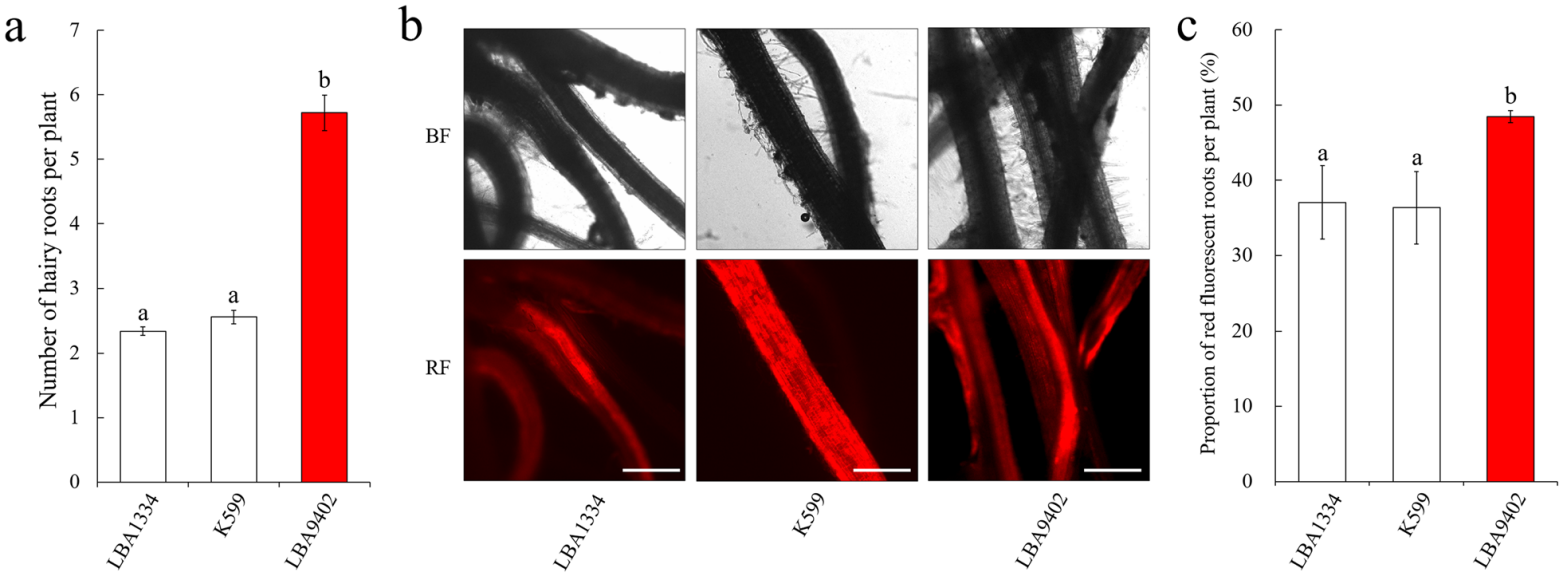
Formation of hairy roots on *L. japonicus* seedlings induced by different *A. rhizogenes* strains carrying pISV-*DsRed1*. The binary vector pISV-*DsRed1* was introduced into the *A. rhizogenes* strains LBA1334, K599 and LBA9402. Each strain was inoculated on 50 plants. Plants were analyzed at 28 dpi. Data indicate means ± SE. Different letters above columns indicate statistically significant differences (Duncan’s Multiple Range test, P < 0.05). **a** Total number of formed hairy roots per plant (n = 50). **b** Microscopic analysis of formed hairy roots under bright field conditions (top) and for red fluorescence (RF) emission (bottom). Bar = 500 μm. **c** Transformation efficiency as determined by the percentage of red fluorescent hairy roots per plant. Data were obtained from plants showing at least one red fluorescent root (n = 8 for LBA1334; n = 20 for K599; n = 23 for LBA9402)

We found in previous studies that pISV2678 is an effective binary vector for *Agrobacterium* transformation. The coding sequence of a given gene can be directly cloned into the multiple cloning site of this vector. To visualize transformed cells in hairy roots of *L. japonicus*, we cloned a *DsRed1* expression cassette into the HindIII site of pISV2678*.* The resulting plasmid, named pISV-*DsRed1,* was then completely sequenced and submitted to the Genbank database (accession number MW701373). A schematic view of the T-DNA of pISV-*DsRed1* is shown in Additional file 2: Figure S1. The binary vector was then mobilized into different *A. rhizogenes* strains (LBA1334, K599 and LBA9402) to test their capacity to transform *L. japonicus* roots. The protocol for hairy root transformation of *L. japonicus* described in this article is based on previously performed experiments [34, 51, 52] and *L. japonicus* transformation protocols from other laboratories [3–12]. Illustrative pictures of the different steps are shown in Fig. 1. Two to eighteen hairy roots reaching a length of 1–4 cm were formed on each plant at 20 dpi. The highest number of hairy roots per plant was observed when LBA9402 (carrying pISV-*DsRed1*) was used for inoculation (Fig. 2a).

Hairy roots expressing *DsRed1* were then identified by fluorescence microscopy. Roots without *DsRed1* expression showed negligible levels of background autofluorescence (Fig. 2b). Plants with at least one red fluorescent root were considered as transgenic. The percentage of obtained transgenic plants varied. When LBA9402 (carrying pISV-*DsRed1*) was used for transformation, 46 ± 4% of the inoculated plants were found to form transgenic hairy roots. A similar transformation frequency was obtained with K599 whereas a considerable reduced value was determined for LBA1334 (20 ± 2%). Significant differences were found for the transformation efficiency as determined by the percentage of transgenic (red fluorescent) roots per plant. Compared to the other strains, LBA9402 (carrying pISV-*DsRed1*) was more efficient in inducing red fluorescent roots (Fig. 2c). Based on these results, LBA9402 was further used for optimization of hairy root transformation.

### Expression of *nopP* increases the transformation efficiency

**Fig. 3:**
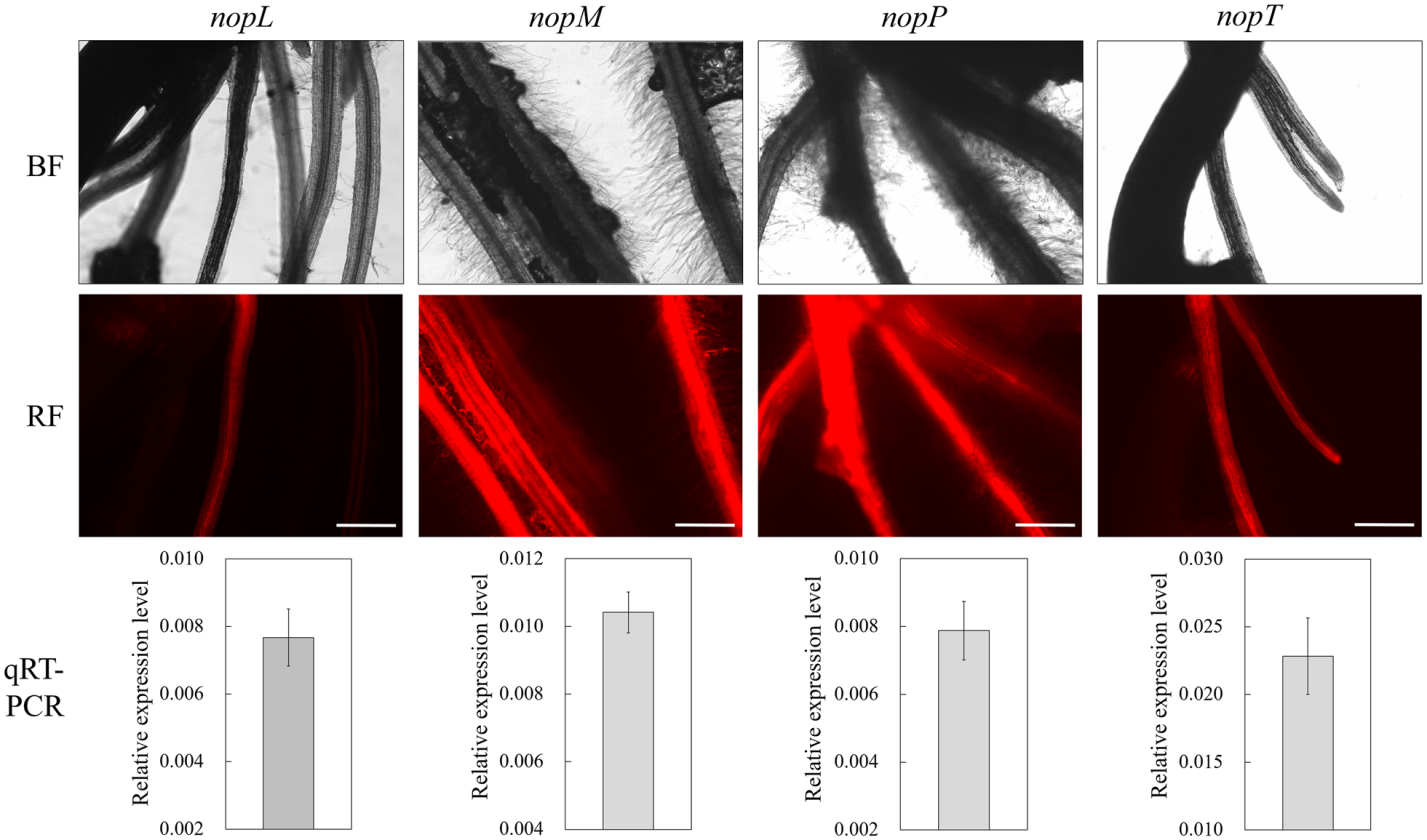
Transformation of *L. japonicus* with pISV-*DsRed1* containing effector genes. The effector genes *nopL*, *nopM*, *nopP* and *nopT* of *Sinorhizobium* sp. NGR234 were cloned into pISV-*DsRed1*. *A. rhizogenes* LBA9402 bacteria carrying the constructed binary vectors were used for transformation. Microscopic analysis of formed hairy roots was performed under bright field conditions (top) and for red fluorescence (RF) emission (bottom) at 28 dpi. Bar = 500 μm. RNA from selected red fluorescent roots was isolated for qRT-PCR analysis to detect effector gene expression (5 plants per RNA extraction). *LjUbiquitin* was used as a reference gene to normalize the transcript abundance value of a given effector gene. Control plants transformed with pISV-*DsRed1* (without effector gene) showed weak background signals in the qRT-PCR analysis. Data indicate means ± SE (n = 3; 3 RNA extractions)

The coding sequences of four T3 effector genes of *Sinorhizobium* sp. NGR234 were cloned into pISV-*DsRed1* to examine effects of these genes on hairy root transformation of *L. japonicus*. The vectors containing *nopL*, *nopM*, *nopP* or *nopT* (pISV-*DsRed1-nopL,* pISV-*DsRed1-nopM,* pISV-*DsRed1-nopP* and pISV-*DsRed1-nopT*, respectively) were mobilized into LBA9402 and the agrobacteria used for hairy root transformation. Microscopic analysis was performed at 28 dpi to detect red fluorescent roots. To confirm effector gene expression, total RNA was extracted from root material showing red fluorescence. Transcripts of *nopL, nopM, nopP,* and *nopT* were detected by qRT-PCR, indicating that the effector genes were co-expressed with *DsRed1* (Fig. 3).

Further microscopic analyses revealed that the strength of red florescence in hairy roots varied. Some roots only showed partially red fluorescence, suggesting that not all root cells were transgenic. Such chimeric roots appeared to be less frequent when pISV-*DsRed1-nopM* or pISV-*DsRed1-nopP* were used for transformation. Furthermore, red fluorescence signals of roots transformed with pISV-*DsRed1-nopM* or pISV-*DsRed1-nopP* appeared sometimes stronger as compared to those obtained with the other vectors.

**Fig. 4:**
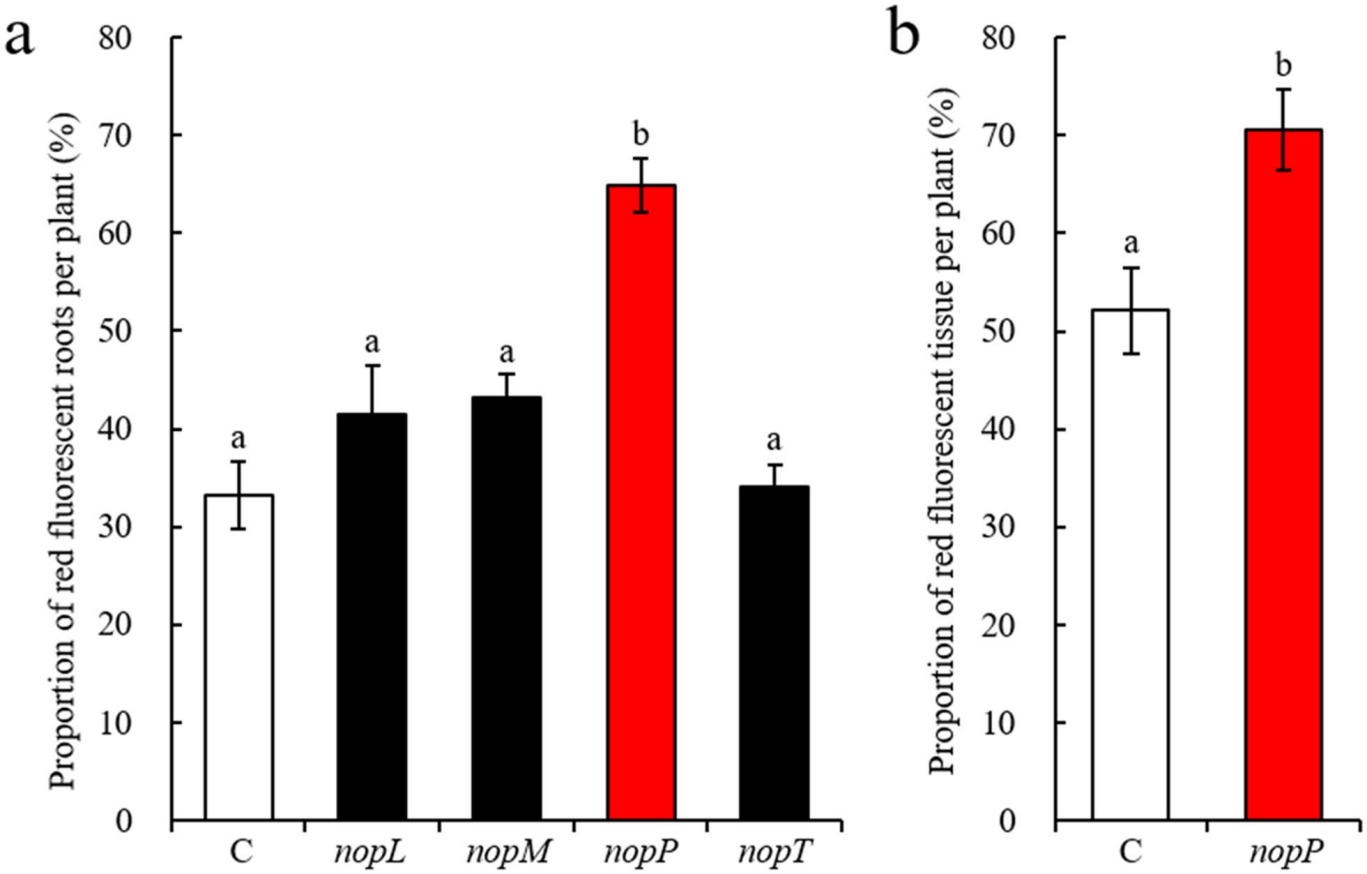
Expression of *nopP* in hairy roots results in an increased transformation efficiency. *L. japonicus* seedlings were transformed with *A. rhizogenes* LBA9402 bacteria carrying pISV-*DsRed1* containing *nopL*, *nopM*, *nopP* and *nopT,* respectively. Plants transformed with pISV-*DsRed1* (without effector gene) served as a control (C). Hairy roots were analyzed for red fluorescence emission at the time of harvest. Histograms indicate means ± SE. Different letters above columns indicate statistically significant differences (Duncan’s Multiple Range test, P < 0.05). **a** Transformation efficiency as determined by the percentage of red fluorescent roots per plant (28 dpi). Each binary vector was examined on 40 plants. Data were obtained from plants showing at least one red fluorescent root (n = 21 for the control (C); n = 18 for *nopL*; n = 15 for *nopM*; n = 22 for *nopP*; n = 16 for *nopT*). **b** Transformation efficiency of control plants (C) and *nopP* expressing plants as determined by the gridline intersection method (45 dpi). Plants showing red fluorescence in at least one hairy root were analyzed (n = 8 for both test groups)

The transformation frequency of plants transformed with binary vectors containing effector genes was then compared to plants transformed with the pISV-*DsRed1* control. In total, each binary vector was examined on 50 plants in 5 test runs. For pISV-*DsRed1,* 56 ± 4% of the inoculated plants were found to form transgenic (red fluorescent) hairy roots. Similar data were obtained for binary vectors containing *nopL* and *nopP *(50 ± 10% and 58 ± 5%, respectively) and lower values for the vectors containing *nopM* and *nopT* (38 ± 10% and 45 ± 6%, respectively). Remarkably, the use of pISV-*DsRed1-nopP* resulted in a significantly increased transformation efficiency as analyzed by the percentage of hairy roots showing red fluorescence. Nearly 70% of roots induced by pISV-*DsRed1-nopP* were fluorescent whereas values for pISV-*DsRed1* and the other vectors were significantly lower (Fig. 4a). Hence, *nopP* expression *in planta* stimulated formation of transgenic hairy roots, while such an effect was not observed for *nopL*, *nopM* or *nopT* expression.

In an additional experiment with pISV-*DsRed1* and pISV-*DsRed1-nopP,* the transformation protocol was slightly modified. After co-culture with *A. rhizogenes,* seedlings were directly placed into the agar plates to develop hairy roots (without use of a filter paper). In this experiment, plants were harvested at 45 dpi and the degree of root tissue showing red fluorescence was determined by the gridline intersection method [50]. Using this method, the ratio of red fluorescent to non-fluorescent tissue was determined for the whole hairy root system independently of the number or size of formed hairy roots. Compared to pISV-*DsRed1,* transformation with pISV-*DsRed1-nopP* resulted in a significant increase of red fluorescent tissue in this experiment (Fig. 4b).

Finally, we used the *A. rhizogenes* strains LBA1334 and K599 to examine the effect of *nopP* on hairy root transformation. Like with LBA9402, pISV-*DsRed1-nopP* was superior to pISV-*DsRed1* when the percentage of hairy roots showing red fluorescence was determined (Additional file 2: Table S3).

### Nodule formation on hairy roots is not affected by *nopP* expression

**Fig. 5:**
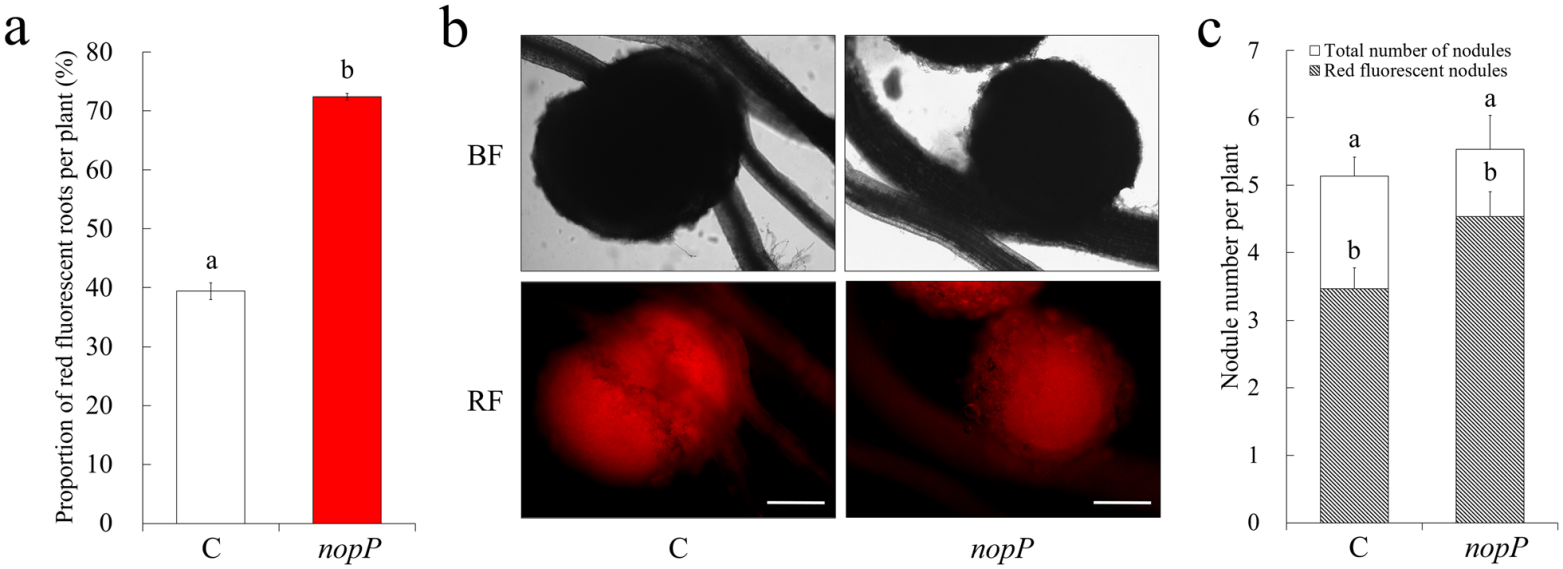
Expression of *nopP* in hairy roots of *L. japonicus* does not affect nodule formation. *A. rhizogenes* LBA9402 carrying the pISV-*DsRed1* control vector or pISV-*DsRed1*-*nopP* was used to induce hairy roots. Data indicate means ± SE. Different letters above columns indicate statistically significant differences between control (C) and *nopP* expressing plants (Duncan’s Multiple Range test, P < 0.05). **a** Transformation efficiency (percentage of red fluorescent roots) for plants showing at least one red fluorescent root (28 dpi). The selected plants (n = 15 for the control (C); n = 18 for *nopP*) were then transferred to jars and inoculated with *M. loti* MAFF303099. **b** Microscopic analysis of formed nodules was performed under bright field conditions (top) and for red fluorescence (RF) emission (bottom) 28 days later. Bar = 500 μm. **c** Total number of nodules per plant and number of nodules showing red fluorescence at the time of harvest

Expression of *nopM* in hairy roots of *L. japonicus* negatively affected nodule formation in a recent study [34]. We therefore wondered whether *nopP* expression also has an impact on nodule formation. LBA9402 carrying either pISV-*DsRed1* or pISV-*DsRed1-nopP* were used for transformation and formed hairy roots were subsequently inoculated with *M. loti* MAFF303099. As in the previous experiments, transformation with pISV-*DsRed1-nopP* analyzed at 28 dpi resulted in a higher percentage of red fluorescent hairy roots as compared with pISV-*DsRed1* (Fig. 5a). Selected transgenic plants (with at least one red fluorescent root) were transferred into plastic jars containing vermiculite and expanded clay. The plants were then inoculated with MAFF303099 and nodule formation was analyzed 28 days later. Most nodules showed red fluorescence (Fig. 5b). The total number of nodules per plant was similar for hairy roots induced by pISV-*DsRed1* and pISV-*DsRed1-nopP.* Due to the higher transformation efficiency, more red fluorescent nodules were counted for plants transformed with pISV-*DsRed1-nopP* as compared to pISV-*DsRed1*. However, this difference was statistically not significant (Fig. 5c). Furthermore, the biomass of nodulated roots was similar for both binary vectors (pISV-*DsRed1*: 3.49 ± 0.32 mg DW per plant; pISV-*DsRed1-nopP*: 3.43 ± 0.23 mg DW per plant). These data indicate that *nopP* expression in host roots did not obviously affect the symbiosis between *L. japonicus* and MAFF303099 under the used experimental conditions.

## Discussion

Hairy root transformation is a powerful technique to rapidly express genes in roots of legumes. In this study, we present a simple and convenient method for *A. rhizogenes*-mediated transformation of the model legume *L. japonicus*. We used *DsRed1* as a marker to ascertain the transgenic nature of obtained hairy roots. Remarkably, we found that *nopP* effector gene expression in *L. japonicus* stimulates formation of transgenic root tissue. Compared to the strains LBA1334 and K599, *A. rhizogenes* LBA9402 was most suitable for hairy root transformation in accordance with published *L. japonicus* transformation protocols [3, 11] and previous work of our laboratory [34, 51, 52]. Strain LBA1334 has been frequently used for *L. japonicus* transformation [5, 8, 10]. Under our experimental conditions, however, LBA1334 carrying pISV-*DsRed1* showed a suboptimal transformation efficiency. K599 was included into our analysis because it has been used for transformation of *Lotus corniculatus* [53]. In our study with *L. japonicus*, induction of transgenic hairy roots by K599 carrying pISV-*DsRed1* was not different from that of LBA1334 and considerably higher than reported previously for K599 containing another binary vector [3].

NopP of *Sinorhizobium* sp. NGR234, an effector originally identified by a phage display approach [54], was found to promote hairy root transformation of *L. japonicus*. In plants transformed with LBA9402 carrying pISV-*DsRed1-nopP*, significantly more red fluorescent roots were obtained as compared to pISV-*DsRed1.* In contrast, *nopL*, *nopM*, and *nopT* did not show such stimulatory effect. The molecular basis for this phenomenon remains to be explored. We suggest that expression of *nopP* in *L. japonicus* suppresses stress-related plant reactions that likely occur in response to *A*. *rhizogenes* inoculation as reported previously [8]. NopP may possess the capacity to suppress defense reactions in *L. japonicus* plants. In line with this hypothesis, a pathogenesis-related protein and a mitogen-activated protein kinase of soybean were identified as putative targets for NopP of *Sinorhizobium fredii* HH103 [55]. Furthermore, TRAPPC13 (trafficking protein particle complex subunit 13-like protein) of *Robinia pseudoacacia* was recently found to interact with NopP of *Mesorhizobium amorphae* CCNWGS0123*.* However, NopP in this legume rather seems to be associated with induction of plant defense responses at an early symbiotic stage [56]. Likewise, expression analysis of pathogenesis-related genes in certain soybeans indicated that strain specific NopP variants stimulate defense gene expression [57, 58]. Remarkably, specific NopP protein variants trigger nodulation blockage depending on the soybean genotype [59, 60]. Two cultivar-specific soybean resistance proteins, GmNNL1 and Rj2 (Rfg1), have been identified to be crucial in this process [58, 61]. GmNNL1 was found to interact directly with NopP of *Bradyrhizobium diazoefficiens* USDA110 [58]. The NopP protein of strain NGR234 used in this study is closely related to NopP proteins produced by *S. fredii* strains forming an incompatible interaction with *Rfg1* soybeans. Based on these findings, we expect that pISV-*DsRed1-nopP* will not be applicable for transformation of all soybean genotypes. Future work will be required to identify effectors that improve hairy root transformation of soybeans and other legumes such as the model legume *Medicago truncatula.*

Our study shows that *nopP* expression in *L. japonicus* does not obviously influence nodule formation. *M. loti* MAFF303099 was used for the nodulation experiment as this strain efficiently nodulates *L. japonicus* and lacks a *nopP* gene in its genome [62]. Prior rhizobial inoculation, transgenic *L. japonicus* seedlings (showing red fluorescence in at least one root) were selected. Considering the fact that *L. japonicus* roots possess strong autofluorescence under green fluorescence conditions, *DsRed1* can be considered as a powerful tool to detect transformed roots. Red fluorescent proteins were also found to be good selection markers for hairy root transformation of other legumes such as soybean [63] and *M. truncatula* [64]. For our nodulation test, hairy roots lacking fluorescence were not removed. Nodule formation on red fluorescent and non-fluorescent roots therefore allowed a direct comparison of transgenic and non-transformed root tissue of a single plant. Using this non-destructive approach, we aimed to keep plant stress, known to negatively affect nodulation, on a minimal level. We also recommend transferring the seedlings to test jars as nodulation on agar plates, although possible, is often suboptimal, perhaps due to ethylene production and other stress factors [8]. Furthermore, to avoid any plant stress, we did not apply antibiotics to eliminate *A. rhizogenes* bacteria.

The genome editing technique CRISPR/Cas9 has been successfully used to knockout specific genes in the *L. japonicus* genome [65]. However, the *A. tumefaciens*-mediated transformation procedure to obtain whole transgenic plants is relatively time-consuming and could be accelerated by the use of *A. rhizogenes* in future. In fact, hairy roots of *L. japonicus* can be regenerated to whole transgenic plants [3]. This opens the possibility to generate mutations in hairy roots using a CRISPR/Cas9 construct. Single red fluorescent hairy roots could be further analyzed for mutations and the *bar* gene expression cassette in the T-DNA region of pISV-*DsRed1* opens the possibility to select for transgenic plants resistant to the herbicide Basta during the shoot induction procedure.

## Conclusion

This article shows that effector genes can be screened for their capacity to improve *A. rhizogenes*-mediated transformation of a given plant species. Expression of the rhizobial effector gene *nopP* in *L. japonicus* roots resulted in a significantly increased transformation efficiency while *nopL*, *nopM*, and *nopT* did not show such an effect. The hairy root transformation protocol for *L. japonicus* described in this article recommends the use of *A. rhizogenes* LBA9402 carrying pISV-*DsRed1-nopP*. The set of constructed pISV-*DsRed1* derivatives containing different effector genes opens the possibility to test effector activities on plant species recalcitrant to *A. rhizogenes* transformation.

## Supplementary Information

**Additional file 1: Table S1.** Strains and plasmids. **Table S2.** Primers used in this study.

**Additional file 2: Figure S1.** Schematic drawing of the T-DNA region of pISV-*DsRed1*. **Table S3.** Effects of *nopP* expression on *L. japonicus* transformation are also observed for *A. rhizogenes* LBA1334 and K599.

## Data Availability

Vectors and *L*. *japonicus* MG-20 seeds can be requested from the corresponding authors. The vectors pISV-*DsRed1* (Addgene ID 171024) and pISV-*DsRed1*-*nopP* (Addgene ID 171025) are also available via Addgene (https://www.addgene.org).
