# Supplementary material for: Use of the rhizobial type III effector gene nopP to improve Agrobacterium rhizogenes-mediated transformation of Lotus japonicus
Source: Plant Methods. 2021 Jun 23;17:66. doi: 10.1186/s13007-021-00764-z (PMC8220826; doi:10.1186/s13007-021-00764-z)
Supplement: Supplementary file 2 — Additional file 2: Figure S1. Schematic drawing of the T-DNA region of pISV-DsRed1. Table S3. Effects of nopP expression on L. japonicus transformation are also observed for A. rhizogenes LBA1334 and K599. [file 13007_2021_764_MOESM2_ESM.pdf]

Additional file 2

Use of the rhizobial type III effector gene *nopP* to improve *Agrobacterium rhizogenes* mediated transformation of *Lotus japonicus*

Yan Wang, Feng Yang, Peng-Fei Zhu, Asaf Khan, Zhi-Ping Xie, Christian Staehelin

This file contains Figure S1 (Schematic drawing of the T-DNA region of pISV-*DsRed1*) and Table S3 (Effects of *nopP* expression on *L. japonicus* transformation are also observed for *A. rhizogenes* LBA1334 and K599).

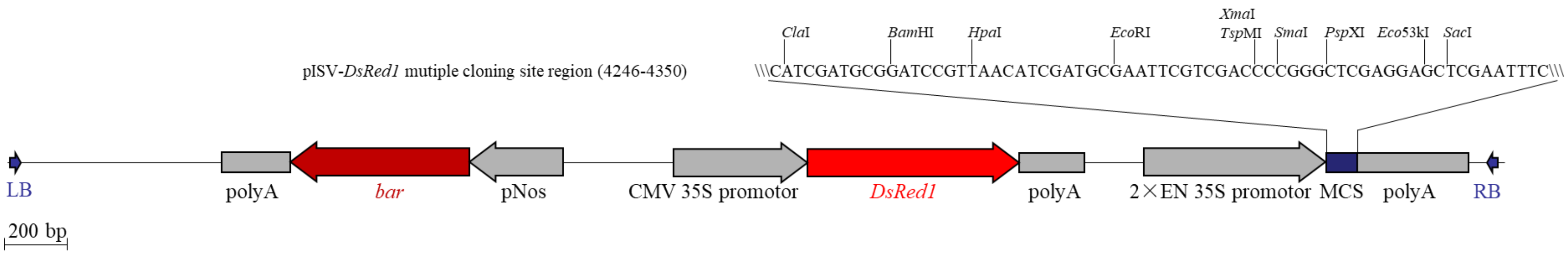

Figure S1 Schematic drawing of the T-DNA region of pISV-*DsRed1*. The 4762-bp T-DNA is flanked by left and right border sequences (LB and RB, respectively). The region contains a *DsRED1* expression cassette for visual selection of transformed plants. In addition, the region contains a *bar* gene expression cassette with a nopaline synthase promoter (possibility to select transgenic plants resistant to the herbicide Basta). In this study, the coding sequences of effector genes were inserted into the multiple cloning site (MCS). Expression of these genes *in planta* is driven by a double cauliflower mosaic virus (CaMV) 35S promoter with a translational enhancer (EN) sequence. All indicated restriction sites in the MCS are unique.

Table S3: Effects of *nopP* expression on *L. japonicus* transformation are also observed for *A. rhizogenes* LBA1334 and K599.

A. rhizogenes	Binary vector	Number of plants*	Transformation efficiency (%)**
LBA1334	pISV-*DsRed1*	8	37.06 ± 4.87§
	pISV-*DsRed1-nopP*	17	45.08 ± 5.22#
K599	pISV-*DsRed1*	20	36.35 ± 4.81§
	pISV-*DsRed1-nopP*	21	44.36 ± 2.53#

* Number of analyzed transgenic plants (showing red fluorescent in at least one hairy root).

** Transformation efficiency as determined by the proportion (%) of red fluorescent roots per plant (28 dpi).

§ Data presented in Fig. 2b.

# Significantly increased values as compared to transformation with pISV-*DsRed1* (Duncan's Multiple Range test, $P<0.05$).
